# A large language model-based tool for identifying relationships to industry in research on the carcinogenicity of benzene, cobalt, and aspartame

**DOI:** 10.1186/s12940-025-01223-1

**Published:** 2025-09-24

**Authors:** Nathan L. DeBono, Vanessa Amar, Hardy Hardy, Mary K. Schubauer-Berigan, Derek Ruths, Nicholas B. King

**Affiliations:** 1 Occupational Cancer Research Centre, Ontario Health, Toronto, Canada; 2 Dalla Lana School of Public Health, University of Toronto, Toronto, Canada; 3 Department of Epidemiology, Biostatistics, and Occupational Health, McGill University, Montreal, Canada; 4 Universitas Mikroskil, Medan, Indonesia; 5 Evidence Synthesis and Classification Branch, International Agency for Research on Cancer, Lyon, France; 6 School of Computer Science, McGill University, Montreal, Canada; 7 Department of Equity, Ethics and Policy, McGill University, Montreal, Canada

## Abstract

**Background:**

Industry-funded research poses a threat to the validity of scientific inference on carcinogenic hazards. Scientists require tools to better identify and characterize industry sponsored research across bodies of evidence to reduce the possible influence of industry bias in evidence synthesis reviews. We applied a novel large language model (LLM)-based tool named InfluenceMapper to demonstrate and evaluate its performance in identifying relationships to industry in research on the carcinogenicity of benzene, cobalt, and aspartame.

**Methods:**

All epidemiological, animal cancer, and mechanistic studies included in systematic reviews on the carcinogenicity of the three agents by the *IARC Monographs* programme. Selected agents were recently evaluated by the *Monographs* and are of commercial interest by major industries. InfluenceMapper extracted disclosed entities in study publications and classified up to 40 possible disclosed relationship types between each entity and the study and between each entity and author. A human classified entities as ‘industry or industry-funded’ and assessed relationships with industry for potential conflicts of interest. Positive predictive values described the extent of true positive relationships identified by InfluenceMapper compared to human assessment.

**Results:**

Analyses included 2,046 studies for all three agents. We identified 320 disclosed industry or industry-funded entities from InfluenceMapper output that were involved in 770 distinct study-entity and author-entity relationships. For each agent, between 4 and 8% of studies disclosed funding by industry and 1–4% of studies had at least one author who disclosed receiving industry funding directly. Industry trade associations for all three agents funded 22 studies published in 16 journals over a 37-year span. Aside from funding, the most prevalent disclosed relationships with industry were receiving data, holding employment, paid consulting, and providing expert testimony. Positive predictive values were excellent (≥ 98%) for study-entity relationships but declined for relationships with individual authors.

**Conclusions:**

LLM-based tools can significantly expedite and bolster the detection of disclosed conflicts of interest from industry sponsored research in cancer prevention. Possible use cases include facilitating the assessment of bias from industry studies in evidence synthesis reviews and alerting scientists to the influence of industry on scientific inference. Persistent challenges in ascertaining conflicts of interest underscore the urgent need for standardized, transparent, and enforceable disclosures in biomedical journals.

**Supplementary Information:**

The online version contains supplementary material available at 10.1186/s12940-025-01223-1.

## Introduction

There is growing recognition of the harmful effects of ‘commercial determinants of health’, defined by the World Health Organization as private sector activities that impact public health and the political and economic systems and norms that enable them [1]. In research, studies funded by industry pose a threat to the validity of scientific inference on carcinogenic hazards. The tobacco industry’s success in undermining scientific evidence of smoking and lung cancer risk [2], which began in the 1950s and continued with the manipulation of scientific discourse of the risks of second-hand smoke through the 1990s [3], has become a powerful and influential model for industries to manipulate, delay, and obfuscate scientific understanding of the carcinogenicity of agents that they have a vested financial interest in being deemed safe. Although the story of tobacco is well known, other known or suspected carcinogens have been subject to similar industry manipulation campaigns, including asbestos [4], lead [5], benzene [6], hexavalent chromium [7], and pesticides [8, 9], among many others [10]. Today, a global product defense industry of law firms, consultants, and paid scientists works to assert the interests of corporate clients in the scientific and regulatory arenas [11]. Studies both within [12–15] and outside [16, 17] the cancer prevention field have substantiated the presence of bias favouring findings of reduced harm in industry-sponsored epidemiological and toxicological studies, and industry’s strategies for manufacturing uncertainty in scientific evidence have also been revealed in internal documents released through litigation to the public [18, 19]. By exploiting and amplifying the uncertainty already inherent in the scientific process, industry has successfully delayed regulation of many agents, with significant consequences for public health [20]. 

Scientists working in the field of cancer prevention have limited tools to deal with this problem. While the International Committee of Medical Journal Editors publishes a standardized form with recommended policies on ‘disclosures of interest’ to promote transparency in scientific publishing, journals employ their own disclosure policies, and funding and conflict of interest information in published articles varies widely in terms of format, types of relationships that are disclosed, extraneous content, and placement in articles. Moreover, disclosure is voluntary, and enforcement of clear and complete disclosure is often impractical. Industry-funded studies in cancer prevention commonly disclose their funding sources, yet declare no conflicts of interest.

Identifying funding and conflicts of interest is generally recommended as a cornerstone of quality and risk of bias assessment in systematic reviews [21]. However, doing so is challenging and labor-intensive, as scientists conducting evidence syntheses may struggle to recognize industry sponsorship, or the impact on bias such sponsorship may entail, particularly from large volumes of studies they are tasked with reviewing. Several authors have developed ‘toolkits’ to help train scientists to better detect and respond to the tactics industry uses to mislead them [22–25]. Given the increasing volume of published articles and complexity of interpreting disclosures, stronger tools are needed to help scientists identify industry influence and minimize bias from commercial interests in evidence syntheses and evaluations.

Large language models (LLM) are increasingly being explored as tools to facilitate systematic reviews in biomedicine [26]. Several studies have applied and evaluated the performance of LLMs in conducting Risk of Bias (ROB) assessments in clinical trials [27–29] and screening of or extracting information from studies in systematic reviews [30, 31]. One study demonstrated substantial accuracy of LLMs in assessing ROB in randomized controlled trials, with mean correct assessment rates (i.e., proportions) exceeding 84% across multiple bias domains compared with human reviewers [27]. The primary strength of LLM-based tools is their ability to dramatically expedite the processing of information from texts that lack rigid structures and exhibit significant variability in language, style, and content. While the performance of LLMs continues to improve, they have already demonstrated efficacy in complementing human review and can relieve some of the extensive time and labour that systematic reviews typically demand. A recently developed LLM-based tool named InfluenceMapper [32], which incorporates a text parser and generative approach for extracting and classifying disclosed relationships to external entities in biomedical studies, is a promising tool for facilitating the surveillance of industry sponsored research. InfluenceMapper is freely available online [33]. 

We conducted the first ever application of InfluenceMapper to identify and characterize relationships to industry in research in cancer prevention. We selected three agents of commercial interest that have recently been reviewed by the International Agency for Research on Cancer’s (IARC) *Monographs* programme on the identification of carcinogenic hazards to humans – benzene, cobalt, and aspartame [34–36]. Using 2,046 epidemiological (i.e., ‘human cancer’), animal cancer, and mechanistic studies included in literature reviews conducted within the *Monographs*, we applied InfluenceMapper to: (1) identify all disclosed relationships between all industry or industry-funded entities and each study and author, (2) evaluate the tool’s performance in identifying true positive relationships with industry entities compared to human assessment, and (3) qualitatively assess identified relationships for conflicts of interest based on the relationship type and the commercial interests of the industry entity. We did not evaluate the tool’s performance in correctly identifying the absence of industry relationships, nor assess the content of studies themselves (i.e., methods, conclusions) in making judgements about the potential for conflicts of interest, as these were beyond the scope of our objectives. InfluenceMapper can streamline and improve the detection of industry-funded research in cancer prevention, facilitate the assessment of bias from industry-funded studies in evidence synthesis reviews, and alert scientists and decision makers to the influence of industry on understandings of the science about known or suspected carcinogenic hazards. Although we focus on cancer, the tool is broadly applicable to all biomedical research on other health hazards and diseases.

## Methods

### Included studies

The analysis included all epidemiological (i.e., ‘human cancer’), animal cancer, and mechanistic studies included in literature reviews by the *IARC Monographs* programme for evidence synthesis evaluations of the carcinogenicity of benzene, cobalt, and aspartame. These agents were chosen because they are of commercial interest by major industries and were recently evaluated by the *Monographs*. The *Monographs* employ systematic and exhaustive searches of literature with studies included according to specific guidelines described in the *Monographs* Preamble [37], with complete search and study screening results for select agents publicly available in the Health Assessment Workspace Collaborative (HAWC) online platform [38, 39]. We identified all included studies from the public HAWC assessments for cobalt and aspartame. For benzene, included studies were all references cited in applicable sections (Sects. “Methods”, “Results”, and “Discussion”) of the *Monographs* volume, as no HAWC assessment was available. Full-text PDFs of included studies were retrieved and matched to their HAWC or bibliographic record using manual and automated matching. A small proportion (6%) of full-text sources (e.g., books, reports) were not retrievable online and were excluded.

### Application of influencemapper

We applied the InfluenceMapper framework tool to the PDFs of all retrieved scientific publications. InfluenceMapper includes a custom fine-tuned GPT-4o-mini LLM trained on 7,861 annotated biomedical publications [32], with scripting to generate final datasets for human analysis. First, we used GROBID [40], an off-the-shelf PDF extractor to parse and retrieve text from each study’s title, author name and affiliation list, and ‘back matter’ sections, which include funding sources, conflicts of interest declarations, acknowledgements, footnotes, and additional author details. GROBID failed completely for 15% of studies, which were typically older studies with slanted, photographic scans of printed articles or poorly structured PDFs. For these studies, PDFs were reviewed by a human and applicable back matter text was extracted manually. All extracted text was then passed to the InfluenceMapper LLM, which inferred relationship information directly from the content of the raw language. In each study, the LLM was prompted to extract: (1) the names of all disclosed entities (i.e., organizations), (2) all disclosed relationships between each entity and the study, and (3) all disclosed relationships between each entity and each author name. The LLM then classified extracted relationships into 12 possible pre-specified study-entity relationship categories and 28 possible pre-specified author-entity relationship categories which are shown in Table S1. Output from the LLM was merged to the HAWC and bibliographic record for each study in preparation for human analysis. A flow diagram of the number of studies included in the workflow is shown in Figure S1.

### Relationships to industry entities

A list of 189 keywords was created to manually identify industry and industry-funded entities from the names of all entities and author affiliations extracted by InfluenceMapper (full keyword list shown in Table S2). An ‘industry or industry-funded entity’ (hereafter referred to as ‘industry entity’) was defined as any non-university, non-governmental organization whose operations are (or have been) financed by commercial businesses or their intermediaries, or who are commercial businesses themselves. To create the keyword list, we first excluded entity names that contained words for academic (i.e., ‘university’) or known government institutions in multiple languages. Then, industry entities were identified using several generic keywords such as ‘Inc.’, ‘Company’, and ‘Consultants’. The remaining entity names were reviewed by author N.D. and industry entity names were added to the keyword list individually. Unrecognized entity names were searched online and classified as ‘industry or industry-funded’ if they reported being a commercial firm or non-governmental organization accepting industry funding or partnerships on a public website. Industry and academic partnerships (e.g., Shell-University of Texas at Austin Unconventional Research Project) were classified as industry entities. All academic and government entity names and all industry and industry-funded entity names selected using the keywords were manually reviewed for validity, and a small number (*n* = 20) of entity names were reclassified. Due to the often vague and generic names of industry-funded research organizations (e.g., Benzene Health Research Consortium), current LLM methods performed poorly at distinguishing these entity names from academic or government institutions, thus necessitating human review.

The performance of InfluenceMapper in correctly identifying relationships that were truly reported in the studies was assessed using the positive predictive value (PPV), a commonly used metric in epidemiology. All study-entity and author-entity relationships identified by InfluenceMapper for industry entities were manually verified by two co-authors (N.D. and V.A.) whose assessment was used as the ‘truth’ for calculation of the tool’s PPV. The PPV was calculated for each relationship type as the number of true positive relationships divided by the total (i.e., true and false) number of positive relationships identified by InfluenceMapper. PPV values ≥ 90% were considered excellent, 70–89% good, 50–69% moderate, and < 50% poor. Relationships were considered false positive if either the entity or author name was incorrect, or if the relationship was not disclosed in the study at all. False positive relationships were excluded from all subsequent analyses.

Descriptive and qualitative analyses were conducted on the full set of studies included in the *Monographs* literature reviews for which a PDF was retrievable. The frequency and proportion of studies disclosing each relationship type overall and according to select characteristics, including evidence type, journal, and publication year, were calculated for each agent. Data were sorted by the number of studies that disclosed each entity name for a given relationship and reviewed to identify prolific industry entities and affiliated authors where the disclosed relationship was considered a potential financial conflict of interest that may have biased the study’s design, results, or interpretation. Relationships were classified as possible sources of bias if the entity was an industry trade association, producer or user of the agent, or a consulting enterprise. Entity names were standardized prior to analyses to account for various spellings or other brand changes over time (e.g., ChemRisk/Cardno ChemRisk). Certain study-entity and author-entity relationships identified by InfluenceMapper were grouped into broader categories to facilitate analyses, such as ‘participating in the design, coordination, analysis, or data collection of the study’ and ‘received travel, speaking, or personal fees or honoraria’. Due to ambiguity in the use of the term ‘support’ as a common synonym for funding, all ‘supported the study’ relationships identified by InfluenceMapper were manually reviewed and a small number (*n* = 3 for cobalt, *n* = 9 for benzene, and *n* = 5 for aspartame) were reclassified to ‘funded the study’ by a human based on subjective judgement using contextual information. All data management and statistical analyses were conducted in R version 4.3.

## Results

A total of 2,046 studies were included in the analysis of all three agents. Among these studies, InfluenceMapper identified a total of 5,941 distinct author-entity and study-entity relationships in the back matter of study publications. From these relationships, the keyword search identified 320 industry entities compared to 1,442 university and government entities and 1,492 entities that had either an uncertain connection to industry or were not identifiable, resulting in a total of 770 disclosed study-entity and author-entity relationships with industry entities. A greater number of industry relationships were identified for aspartame (*n* = 321) compared to benzene (*n* = 255) or cobalt (*n* = 194) (Table 1). Positive predictive values were excellent for nearly all study-entity relationships (≥ 89%) and virtually perfect for study-entity relationships that pertained to industry funding the study. Positive predictive values were lower for author-entity relationships, with false positives common for relationships involving an author’s direct receipt of research funds. Among all studies, GROBID failed to extract author affiliations for approximately half (45–63%) of the 9,368 distinct author names, although 81 author affiliations were still identified as industry entities across all three agents. A total of 52 studies had at least one author with an industry affiliation who did not disclose any author-entity relationships in the article. A complete data set of all study information and relationships shown in Table 1 is provided in supplemental material.

**Fig. 1:**
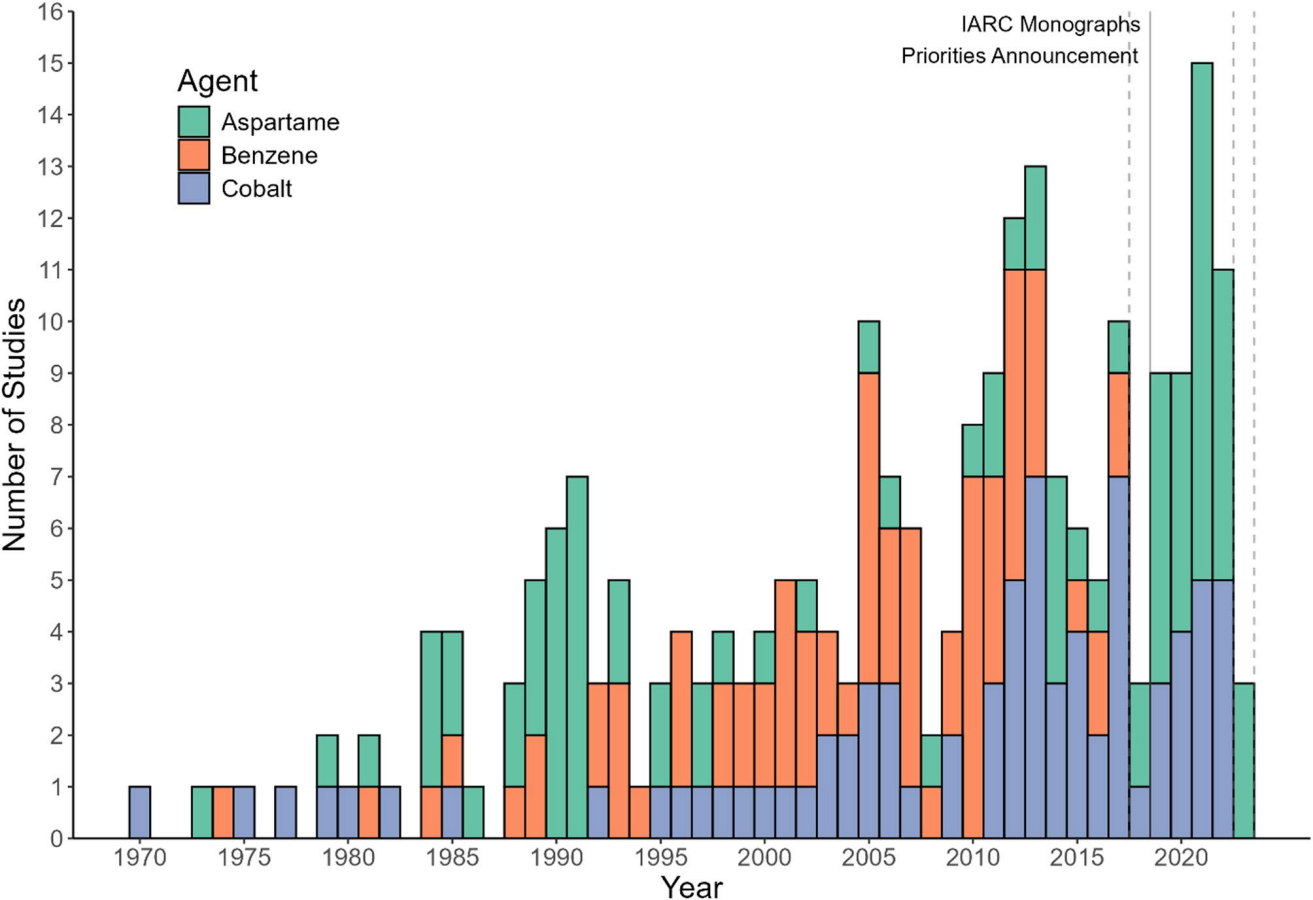
Number of studies published with any disclosed relationship to industry entities by calendar year from systematic reviews by the *IARC Monographs* programme of the carcinogenicity of benzene, cobalt, and aspartame. * Vertical lines correspond to the year of monograph publication for benzene (2018), cobalt (2023), and aspartame (2024), as well as the IARC Advisory Group’s recommended priorities (2019). The priorities announcement in 2019 identified aspartame and cobalt as agents with a high priority for evaluation within the subsequent 5 years. † ‘Industry entity’ was defined as any non-university, non-governmental organization whose operations are (or have been) financed by commercial businesses or their intermediaries, or who are commercial businesses themselves

The number and proportion of studies that disclosed any relationship to industry entities is shown in Table 2. For benzene, cobalt, and aspartame, there were 55, 72, and 59 such studies, respectively. The most commonly disclosed relationship between industry entities and the studies was funding, with between 4 and 8% of studies funded by industry across the three agents. The next most frequent relationship involved supplying data or materials to the study, although some of these were acknowledgements to companies for supplying assay or reagent materials in laboratory experiments. For author-entity relationships, the most frequently disclosed relationship was receiving direct research funds, with consulting and expert testimony relationships also frequently disclosed in studies for benzene and aspartame. In total, between 7 and 14% of studies disclosed any study-entity or author-entity relationship with an industry entity, with these studies predominantly being mechanistic studies compared to the other evidence types. Industry related research was most frequently published in Environmental Health Perspectives, the Journal of Occupational and Environmental Medicine, the American Journal of Clinical Nutrition, Chemical-Biological Interactions, and Regulatory Toxicology and Pharmacology. An illustration of the number of studies with any disclosed relationship to industry published per year is shown in Fig. 1. Notably, a significant number of studies for aspartame were published after the 2019 IARC Advisory Group recommended it with high priority for evaluation, indicating potential interest from industry entities with a commercial stake in the evaluation.

Select studies with relationships to industry entities considered to be potential sources of bias are presented for each agent in Tables 3, 4 and 5. For benzene and cobalt, the most prolific entities funding studies were industry trade associations representing the oil and gas and metal mining and production industries, namely the American Petroleum Institute and the Cobalt Institute/Cobalt Development Institute. For aspartame, funding by direct producers or users of the chemical was more common, although trade associations for the beverage and artificial sweetener industries, the American Beverage Association and the Calorie Control Council, funded three studies. Consulting firms disclosed as participating in conducting studies, or as the affiliations of authors who disclosed employment, consulting, or expert testimony for industry entities, included Exponent, ToxStrategies, and ChemRisk/Cardno ChemRisk. For benzene, a significant number of studies disclosed one or more authors from the University of California, Berkeley who provided expert testimony for defendants in legal cases involving benzene exposure.

## Discussion

Using InfluenceMapper, we demonstrated nearly perfect positive predictive value in identifying a significant number of studies that were funded by industry or entities with commercial interest in research on the carcinogenicity of benzene, cobalt, and aspartame. Although human effort was required, the tool dramatically expedited the identification and characterization of relationships to industry in entire bodies of evidence for three distinct agents. The assessment of disclosed relationships revealed a modest prevalence of studies with a financial, material, or professional relationship to specific industry organizations responsible for seeding the literature with studies that were conducted with a potential conflict of interest and risk of bias. We emphasize that InfluenceMapper does not determine whether a given relationship constitutes a conflict of interest, with some of the identified relationships to industry (e.g., receiving technical assistance) providing no indication of a competing interest over the study’s results. Instead, it greatly facilitates critical review and expert judgement regarding which relationships warrant concern for a given scientific objective.

While InfluenceMapper showed good or excellent positive predictive values for relationships other than author funding, our estimates are conservative and likely constitute a lower bound of the prevalence of studies with relationships to industry. First, author disclosures are voluntary, and there is evidence from clinical epidemiology studies that authors frequently fail to disclose relevant relationships in published articles [41]. Second, given the wide variability and sometimes intentional ambiguity in how industry relationships are disclosed in scientific articles (particularly in the past, when disclosure policies may have been laxer), and the inability to identify many entity names, a significant number of studies with relationships to industry may have been missed. Despite these limitations, our findings underscore an opportunity for InfluenceMapper to be used as a powerful tool to promote greater transparency and objectivity in cancer prevention research and evidence synthesis reviews.

This is to our knowledge the first study to systematically evaluate research involving commercial interests, including industry funding, on health hazards using a large language model. While numerous reviews and commentaries have been published about industry influence on research on environmental and occupational hazards [42–51], a smaller number of studies have evaluated the problem through systematic empirical assessments of literature [12, 13, 15, 52]. The two largest studies to date that have systematically analyzed conflict of interest or industry funding disclosures in studies on environmental and occupational hazards included a total of 510 and 373 articles [12, 15], with the volume of studies presumably limited by the intensive manual labor required for text review, extraction, and interpretation. In the present work, we analyzed 2,046 studies with relative ease, highlighting the dramatically expedited workflow made possible with tools like InfluenceMapper. Although previous assessments have focused on industry bias as outcomes of interest, InfluenceMapper extracts all entities and relationships disclosed in scientific articles and is largely agnostic to whether these outputs constitute conflicts of interest or introduce bias in study results. These objectives are accomplished only by users of InfluenceMapper output, and we demonstrated how the tool can be used for this purpose to greatly accelerate the process of identifying studies suspected to be biased by commercial interests as shown in Tables 3, 4 and 5.

For all three agents, the number of studies that disclosed any relationship to industry entities was modest when expressed as a proportion of the full number of studies included in the epidemiology (i.e., ‘human cancer’), animal cancer, and mechanistic evidence sections of the *Monographs* literature reviews. Despite this, our finding that between 6 and 8% of studies are funded by industry is consistent with a previous analysis of disclosed industry funding in all occupational cohort studies of cancer risk published between 2000 and 2010, which reported a prevalence of 8%.^12^ While these proportions are modest, they should not be interpreted as indicating a minor influence of industry on the body of scientific evidence for these agents. Small numbers of industry-funded studies may succeed in undermining expert opinion by exploiting and amplifying the uncertainty that is already inherent in the process of scientific inquiry, which can have a downgrading effect on evaluations of carcinogenicity conducted by agencies like the U.S. National Toxicology Program, European Food Safety Agency, and IARC [53, 54]. 

Despite the potential utility of InfluenceMapper, key limitations remain with the current technology and its application to identifying industry relationships in research. First, the identification of industry entities from the total list of entities extracted by InfluenceMapper required human effort and relied on the availability of external information from publicly accessible sources. The names of industry-funded research entities are often misleading or their connection to entities with political or commercial interests may be obscured. For example, the Center for Truth in Science is an organization supporting research on the carcinogenicity of ethylene oxide, formaldehyde, glyphosate (i.e., ‘RoundUp’ herbicide), perfluoroalkyl substances (PFAS), and talcum powder, but does not transparently disclose sources of funding [55]. However, these challenges are not unique to InfluenceMapper itself and would be still be present, and likely be even more time consuming to address, had our analysis been conducted exclusively by humans. Second, the number of false positive relationships with individual authors identified by the GPT-4o mini LLM was significant and human effort is still required to check the validity of these relationships from the tool’s output. As the LLM’s responses are probabilistic, responses may also change with each instance the model is prompted to interpret the same text. Further, the parsing of text for the institutional affiliation of authors by the GROBID machine learning model often failed, resulting in a high proportion of missing affiliations in InfluenceMapper output. This is a meaningful loss of information, as studies published by industry authors or paid consultants may be missed if they do not disclose other industry relationships in the back matter of the article. While LLM and optical character recognition technology is likely to improve, the ambiguity and lack of transparency in disclosed entities and their relationships to the research will persist without changes to the norms of publishing practices in the field, such as standardized disclosure requirements and stricter enforcement by journals. Transparency in conflicts of interest would be enhanced by journals adopting a universal principle of reporting all funding and other potential conflicts of interest in the publicly available metadata for each manuscript, regardless of its open-access licensing status.

Despite these challenges, we envision three viable use cases of InfluenceMapper to investigate and address the influence of commercial interests in research evaluating carcinogenic hazards, or any other health hazard or disease in biomedicine. First, the tool can be used to improve the detection of industry funded research and consultants in the field and foster transparency in evidence synthesis reviews. Discerning industry relationships from disclosure statements in scientific articles and evaluating their potential to pose meaningful conflicts of interest, is often a complex and time-consuming process, particularly when reviewing a large number of studies is required. Scientists typically lack a systematic understanding of the nature and extent of industry-funded research across a complete body of evidence or are uninformed about which authors and entities are prolific. Further, bodies that are required to vet scientists for conflicts of interest or scrutinize disclosure statements, such as evidence synthesis review panels or editors of scientific journals, are limited by practical difficulties in verifying an author’s independence from specific industries that may be prohibited, such as the tobacco or alcohol industries [56, 57]. The speed with which tools like InfluenceMapper can extract and synthesize information creates powerful opportunities to apply the tool systematically across evidence bases for widespread surveillance of industry research activities in a given field.

Second, InfluenceMapper can facilitate qualitative and quantitative assessments of bias from industry-funded studies in evidence synthesis reviews. LLMs have already been used to expedite ROB assessments in systematic reviews of results from clinical trials with remarkable accuracy [27]. Given the continually growing number of scientific publications each year, tools like InfluenceMapper will become increasingly useful in accelerating the extraction and classification of industry relationships from publications, allowing studies to be grouped and compared according to their sources of funding or other disclosed relationships, as is routinely done for other sources of study bias, such as confounding. By comparing industry-funded studies to those free from conflicts of interest, either qualitatively or empirically through stratified meta-analyses, scientists can more easily make their own judgements regarding the magnitude and direction of bias introduced by industry-funded research in evidence syntheses, hazard evaluations, and risk assessments. Recommendations have been proposed for formally recognizing financial conflicts of interest as a potential source of bias in the Cochrane Handbook for Systematic Reviews and in evidence synthesis evaluations conducted by U.S. and European organizations, as well as IARC [21, 54, 58]. 

Third, insights gained from evaluations of industry-funded research may alert scientists to industry activities and train them in the tactics industry uses to delay, mislead, and obstruct scientific consensus. Particularly for early-career scientists new to the cancer prevention field, increased awareness of journals, institutions, and authors who frequently publish industry-funded research is a critical first step for protecting personal scientific judgements from being undermined by commercial interests. For example, awareness of our finding that Regulatory Pharmacology and Toxicology and the Journal of Occupational and Environmental Medicine, two journals with a documented history of controversial publishing practices or connections to industry [7, 14, 59– 61], frequently published industry-supported research on benzene and cobalt may increase scrutiny over studies from those journals. As industry may seek to obscure their influence on research and scientific discourse, evidence-based insights characterizing the nature of industry research can bolster the training scientists receive to minimize bias in their assessments of evidence and protect the integrity of their discipline.

## Conclusion

We demonstrated that an LLM-based tool is effective for expediting the extraction and interpretation of industry relationships and conflicts of interest in large bodies of research on carcinogenic hazards. We estimated the prevalence of study and author relationships to industry and industry-funded entities and identified prolific industry groups that have supported numerous publications in select journals on the carcinogenicity of benzene, cobalt, and aspartame. Our results underscore the urgent need for more standardized, transparent, and enforceable conflict of interest and funding disclosures in biomedical journals. Our proposed use cases for tools like InfluenceMapper may better protect the scientific enterprise from industry manipulation and bias.

**Table 1:** Number of true positives and positive predictive value (PPV^*^) for industry relationships identified in the influencemapper analysis of systematic reviews by the *IARC monographs* programme of the carcinogenicity of benzene, cobalt, and aspartame

	Benzene (2018)		Cobalt (2023)		Aspartame (2024)	
	No.	PPV %	No.	PPV %	No.	PPV %
Disclosed relationship of study to industry entities†						
Funded the study	49	100	59	100	40	98
Reviewed the study	1	100	14	100	3	100
Supplied data or materials to the study	14	100	15	100	25	100
Supported the study	13	100	13	100	6	100
Participated in the design, coordination, analysis, or data collection of the study	12	92	8	89	6	100
Disclosed relationship of author to industry entities						
Received research grant	126	83	22	59	55	54
Hired as consultant	5	100	7	100	54	87
Provided expert testimony	12	100	2	100	1	100
Received travel, speaking, or personal fees or honoraria	-	-	-	-	79	96
Held employment, board, or equity position	20	100	51	100	43	98
Received academic award, scholarship, or appointment	3	33	3	75	9	82
All disclosed industry relationships total	255	-	194	-	321	-
Author affiliations that were industry entities‡	24	-	44	-	13	-

Abbreviations: No., Number

* Positive predictive value is the proportion of positive relationships identified by InfluenceMapper that were correct. It is calculated as the number of true positive relationships divided by the total number of true and false positive relationships identified with industry or industry-funded entities

† ‘Industry entity’ was defined as any non-university, non-governmental organization whose operations are (or have been) financed by commercial businesses or their intermediaries, or who are commercial businesses themselves

‡ Among all 2,046 studies analyzed, the author affiliation was missing for 50% of 2,227 distinct author names for benzene, 45% of 4,550 distinct author names for cobalt, and 63% of 2,591 distinct author names for aspartame

**Table 2:** Frequency of studies with disclosed relationships to industry entities from systematic reviews by the *IARC monographs* programme of the carcinogenicity of benzene, cobalt, and aspartame

	Benzene (2018)	Cobalt (2023)	Aspartame (2024)
	No. of studies (%) *		
Disclosed relationship of study to industry entities†			
Funded the study	39 (8)	47 (4)	39 (6)
Reviewed the study	1 (0)	5 (0)	2 (0)
Wrote the study	0	0	0
Supplied data or materials to the study	10 (2)	13 (1)	15 (2)
Supported the study ‡	9 (2)	10 (1)	5 (1)
Participated in the design, coordination, analysis, or data collection of the study	10 (2)	8 (1)	6 (1)
Total	55 (11)	72 (7)	59 (10)
Disclosed relationship of author to industry entities			
Received research grant	20 (4)	6 (1)	17 (3)
Received research materials	0	0	0
Hired as consultant	5 (1)	5 (0)	14 (2)
Provided expert testimony §	11 (2)	2 (0)	1 (0)
Received travel, speaking, or personal fees or honoraria	0	0	10 (2)
Held employment, board, or equity position	4 (1)	14 (1)	16 (3)
Received academic award, scholarship, or appointment	2 (1)	3 (0)	4 (1)
Total	33 (6)	21 (2)	38 (6)
Any disclosed industry relationship total	75 (15)	78 (7)	84 (14)
Evidence type(s) ††			
Epidemiological (i.e., human cancer)	18	13	11
Animal cancer	0	1	9
Mechanistic	57	65	69

	Journal name (No. of studies)		
Most frequent journals of publication, any disclosed industry relationship	Environ Health Perspect (11)	Regul Toxicol Pharmacol (6)	Am J Clin Nutr (9)
	Chem-Biol Interact (7)	J Occup Environ Med (5)	Nutrients (6)
	Carcinogenesis (7)	J Biol Chem (3)	Physiol Behav (6)
	Occup Environ Med (5)	Environ Pollut (2)	Metabolism (5)
	J Occup Environ Med (4)	J Environ Monit (2)	Food Chem Toxicol (4)

Abbreviations: No., Number

* Total number of epidemiological (i.e., ‘human cancer’), animal cancer, and mechanistic studies included in the literature review by the *IARC Monographs* programme was 1,062 for cobalt, 509 for benzene (based on citations in the *Monographs* volume only), and 619 for aspartame

† ‘Industry entity’ was defined as any non-university, non-governmental organization whose operations are (or have been) financed by commercial businesses or their intermediaries, or who are commercial businesses themselves

‡ Excludes studies where ‘support’ was considered a synonym for funding rather than expert or technical assistance

§ Includes studies where expert testimony was disclosed for defendants but an industry entity name was not specified

†† Epidemiological evidence types are studies of cancer in humans. Mechanistic evidence may also include epidemiological studies of mechanistic outcomes

**Table 3:** Entities and author affiliations in select^*^ studies with disclosed relationships to industry considered potential conflicts of interest from the systematic review by the *IARC monographs* programme of the carcinogenicity of benzene

	No. studies†	Publication year(s)	Journal(s)	Evidence type(s)‡
Industry entities with disclosed relationship to study§				
Funded the study				
American Petroleum Institute	10	1985–2016	J Occup Environ Med, Carcinogenesis, J Natl Cancer Inst, J Toxicol Environ Health A, Cell Biol Toxicol, Occup Environ Med, Mol Pharmacol, Environ Health Perspect, Am J Ind Med	Epidemiological, Mechanistic
Benzene Health Research Consortium	5	2005–2010	Chem-Biol Interact, Leuk Res	Epidemiological, Mechanistic
Health Effects Institute	4	1999–2002	Cancer Res, Am J Ind Med, Mutat Res, Res Rep Health Eff Inst	Mechanistic
Energy Institute/Institute of Petroleum	4	1993–2012	J Natl Cancer Inst, Occup Environ Med, Occup Med, Environ Health Perspect	Epidemiological
ExxonMobil	3	2001–2010	Chem-Biol Interact, J Occup Environ Med	Mechanistic, Epidemiological
American Chemistry Council	2	2005–2008	J Toxicol Environ Health A, Chem-Biol Interact	Mechanistic
Concawe	1	2017	Chem-Biol Interact	Mechanistic
Australian Institute of Petroleum	1	2012	J Natl Cancer Inst	Epidemiological
Canadian Petroleum Products Institute	1	2012	J Natl Cancer Inst	Epidemiological
Dow Chemical	1	2010	Chem-Biol Interact	Mechanistic
Imperial Oil	1	2003	Occup Environ Med	Epidemiological
Supplied data to the study				
Chevron, ExxonMobil, Shell	1	1999	Occup Environ Med	Epidemiological
American Petroleum Institute	1	1989	Environ Health Perspect	Mechanistic
Participated in conducting the study				
Industry Dynamics Associates	2	2001	J Occup Environ Med	Epidemiological
American Petroleum Institute	1	2010	Chem-Biol Interact	Epidemiological
Exponent	1	2003	J Toxicol Environ Health A	Epidemiological
Neste Group	1	1998	J Occup Environ Med	Epidemiological
Affiliations of authors with disclosed relationship to industry entities††				
Received research funding				
University of California Berkeley, U.S.	4	2011–2013	Carcinogenesis, Environ Mol Mutagen, Environ Health Perspect	Mechanistic
University of Birmingham, U.K.	1	2005	Occup Environ Med	Epidemiological
Held employment				
ExxonMobil, U.S.	2	2010–2012	J Natl Cancer Inst, Chem-Biol Interact	Epidemiological, Mechanistic
Dow Chemical, Netherlands	1	2010	Chem-Biol Interact	Mechanistic
Provided expert testimony				
University of California Berkeley, U.S.	9	2005–2016	Carcinogenesis, Leukemia, Chem-Biol Interact, Environ Health Perspect, Pharmacogenet Genom	Mechanistic

Abbreviations: No., Number

* Selected studies are those where a disclosed relationship to an industry or industry-funded entity was considered a conflict of interest because it may have biased the study’s analysis, results, or conclusions. Bias was considered possible if the entity was an industry trade association, producer or user of the agent, or a consulting enterprise. Citation information for each study is available in data set in supplemental material

† Multiple entity or author relationships may be disclosed in a single publication. Study counts are not mutually exclusive

‡ Epidemiological evidence types are studies of cancer in humans. Mechanistic evidence may also include epidemiological studies of mechanistic outcomes

§ ‘Industry entity’ was defined as any non-university, non-governmental organization whose operations are (or have been) financed by commercial businesses or their intermediaries, or who are commercial businesses themselves

†† Text parsing of author institutional affiliation failed for 45 distinct author names in a total of 18 of 30 studies that disclosed any author-industry entity relationship

**Table 4:** Entities and author affiliations in select^*^ studies with disclosed relationships to industry considered potential conflicts of interest from the systematic review by the *IARC monographs* programme of the carcinogenicity of Cobalt

	No. studies†	Publication year(s)	Journal(s)	Evidence type(s)‡
Industry entities with disclosed relationship to study§				
Funded the study				
Cobalt Institute/Cobalt Development Institute	9	2000–2022	Regul Toxicol Pharmacol, Toxicol Sci, Occup Environ Med, Toxicol Lett, J Environ Monit, Environ Mol Mutagen	Mechanistic
Cobalt REACH Consortium	5	2015–2022	Regul Toxicol Pharmacol, Toxicol Sci	Mechanistic
DePuy Orthopedics/International	5	2011–2016	Oncotarget, Crit Rev Toxicol, Am J Clin Nutr, Food Chem Toxicol, Toxicol In Vitro	Mechanistic
International Tungsten Industry Association	4	2017	J Occup Environ Med	Epidemiological
MedTech Europe	2	2021–2022	Regul Toxicol Pharmacol	Epidemiological
IBM Corporation	1	2020	Occup Environ Med	Epidemiological
Precision Castparts Corporation	1	2019	Sci Total Environ	Mechanistic
Freeport Cobalt	1	2017	BMC Cancer	Epidemiological
Swedish ‘hardmetal industry’	1	2017	J Occup Environ Med	Epidemiological
Nickel Producers Environmental Research Association (NiPERA)	1	1999	J Environ Monit	Mechanistic
Reviewed the study				
Johnson & Johnson	2	2021–2022	Regul Toxicol Pharmacol	Epidemiological
EBRC Consulting	1	2015	Regul Toxicol Pharmacol	Mechanistic
IBM Corporation	1	2020	Occup Environ Med	Epidemiological
Supplied data to the study				
IBM Corporation	1	2020	Occup Environ Med	Epidemiological
Affiliations of authors with disclosed relationship to industry††				
Received research funding				
Cobalt Institute, U.K.	2	2022	Regul Toxicol Pharmacol	Mechanistic
Eurométaux, Belgium	1	2022	Regul Toxicol Pharmacol	Mechanistic
Nickel Producers Environmental Research Association (NiPERA), U.S.	1	2022	Regul Toxicol Pharmacol	Mechanistic
Williams Chemical Consultancy, Ireland	1	2020	Toxicol Sci	Mechanistic
Held employment				
ChemRisk/Cardno ChemRisk, U.S.	4	2013–2022	Regul Toxicol Pharmacol, Crit Rev Toxicol	Epidemiological, Mechanistic
Umicore, Belgium	3	2011–2020	Toxicol Sci, Occup Environ Med	Mechanistic
Cobalt Institute, U.K.	3	2020–2022	Regul Toxicol Pharmacol, Toxicol Sci	Mechanistic
LifeScan Corporation, U.S.	1	2021	Regul Toxicol Pharmacol	Mechanistic
Nickel Producers Environmental Research Association (NiPERA), U.S.	1	2022	Regul Toxicol Pharmacol	Mechanistic
Hired as consultant				
ChemRisk/Cardno ChemRisk, U.S.	1	2015	Crit Rev Toxicol	Mechanistic
Kirkland Consulting, U.K.	1	2015	Regul Toxicol Pharmacol	Mechanistic
Provided expert testimony				
ChemRisk/Cardno ChemRisk, U.S.	2	2013–2015	Crit Rev Toxicol, J Toxicol Environ Health A	Mechanistic

Abbreviations: No., Number

* Selected studies are those where a disclosed relationship to an industry or industry-funded entity was considered a conflict of interest because it may have biased the study’s analysis, results, or conclusions. Bias was considered possible if the entity was an industry trade association, producer or user of the agent, or a consulting enterprise. Citation information for each study is available in data set in supplemental material

† Multiple entity or author relationships may be disclosed in a single publication. Study counts are not mutually exclusive

‡ Epidemiological evidence types are studies of cancer in humans. Mechanistic evidence may also include epidemiological studies of mechanistic outcomes

§ ‘Industry entity’ was defined as any non-university, non-governmental organization whose operations are (or have been) financed by commercial businesses or their intermediaries, or who are commercial businesses themselves

†† Text parsing of author’s institutional affiliation failed for 18 distinct author names in a total of 7 of 21 studies that disclosed any author-industry entity relationship

**Table 5:** Entities and author affiliations in select^*^ studies with disclosed relationships to industry considered potential conflicts of interest from the systematic review by the *IARC monographs* programme of the carcinogenicity of aspartame

	No. studies†	Publication year(s)	Journal(s)	Evidence type(s)‡
Industry entities with disclosed relationship to study§				
Funded the study				
NutraSweet Company	10	1998–2000	Food Chem, Ann NY Acad Sci, Am J Gastroenterol, Epilepsy Res, Pharmacol, J Neurochem, Am J Clin Nutr, Metabolism, Diabetes	Mechanistic
International Life Sciences Institute	6	1986–2014	Am J Clin Nutr, Physiol Behav, Metabolism, Food Chem Toxicol	Mechanistic
G.D. Searle	4	1979–1989	Arch Intern Med, Diabetes Care, J Nutr	Animal bioassay, Mechanistic
Calorie Control Council	2	2019–2021	Environ Health, Regul Toxicol Pharmacol	Animal bioassay
PepsiCo	2	2014–2020	Nutrients, Chem-Biol Interact	Mechanistic
American Beverage Association	1	2023	Food Chem Toxicol	Animal bioassay
Reviewed the study				
American Beverage Association	1	2023	Food Chem Toxicol	Animal bioassay
Calorie Control Council	1	2019	Regul Toxicol Pharmacol	Animal bioassay
Participated in conducting the study				
ToxStrategies	1	2023	Food Chem Toxicol	Animal bioassay
NutraSweet Company	1	1989	Arch Int Med	Mechanistic
Affiliations of authors with disclosed relationship to industry††				
Held employment				
ToxStrategies, U.S.	1	2023	Food Chem Toxicol	Animal bioassay
Nutrition Impact, U.S.	1	2022	Nutrients	Epidemiological
Ajinomoto Company, Japan	1	2019	Regul Toxicol Pharmacol	Animal bioassay, Mechanistic
PepsiCo, U.S.	1	2014	Chem Biol Interact	Mechanistic
Hired as consultant				
University of Washington, U.S.	1	2022	Nutrients	Epidemiological
University of Bristol, U.K.	1	2021	Int J Obes	Mechanistic
University of Southampton, U.K.	1	2008	Food Chem Toxicol	Mechanistic

Abbreviations: No., Number

* Selected studies are those where a disclosed relationship to an industry or industry-funded entity was considered a conflict of interest because it may have biased the study’s analysis, results, or conclusions. Bias was considered possible if the entity was an industry trade association, producer or user of the agent, or a consulting enterprise. Citation information for each study is available in data set in supplemental material

† Multiple entity or author relationships may be disclosed in a single publication. Study counts are not mutually exclusive

‡ Epidemiological evidence types are studies of cancer in humans. Mechanistic evidence may also include epidemiological studies of mechanistic outcomes

§ ‘Industry entity’ was defined as any non-university, non-governmental organization whose operations are (or have been) financed by commercial businesses or their intermediaries, or who are commercial businesses themselves

†† Text parsing of author’s institutional affiliation failed for 40 distinct author names in a total of 26 of 38 studies that disclosed any author-industry entity relationship

## Supplementary Information

Below is the link to the electronic supplementary material.

Supplementary Material 1.

Supplementary Material 2.

## Data Availability

The datasets generated and/or analysed during the current study are available in supplemental material.
