# Supplementary material for: A large language model-based tool for identifying relationships to industry in research on the carcinogenicity of benzene, cobalt, and aspartame
Source: Environ Health. 2025 Sep 24;24:64. doi: 10.1186/s12940-025-01223-1 (PMC12462328; doi:10.1186/s12940-025-01223-1)
Supplement: Supplementary file 2 — Supplementary Material 2. [file 12940_2025_1223_MOESM2_ESM.docx]

**Table S1. Possible entity relationship types classified by the InfluenceMapper custom GPT-4o-mini large language model**

| **Study-entity relationships** | |
| --- | --- |
|  | Perform analysis |
|  | Collect data |
|  | Coordinate the study |
|  | Design the study |
|  | Fund the study |
|  | Participate in the study |
|  | Review the study |
|  | Supply the study |
|  | Supply data to the study |
|  | Support the study* |
|  | Write the study |
|  | Other |
| **Author-entity relationships** | |
|  | Honorarium |
|  | Named professor |
|  | Received research materials directly |
|  | Patent license |
|  | Personal fees |
|  | Salary support |
|  | Received research materials indirectly |
|  | Equity |
|  | Expert testimony |
|  | Consultant |
|  | Board member |
|  | Founder of entity or organization |
|  | Received travel support |
|  | Holds chair |
|  | Fellowship |
|  | Scholarship |
|  | Collaborator |
|  | Received research grant funds directly |
|  | Speakers’ bureau |
|  | Employee of |
|  | Received research grant funds indirectly |
|  | Patent |
|  | Award |
|  | Research trial committee member |
|  | Supported |
|  | Former employee of |
|  | Other/unspecified |
|  | No relationship |
|  |  |

* The LLM interprets ‘support’ relationships verbatim and relationships could entail either financial funding or expressions of gratitude for expert or technical assistance. Some support relationships were re-classified by the authors as described in the main text.

**Figure S1. Inclusion of studies in the InfluenceMapper analysis of IARC Monographs literature reviews for the carcinogenicity of benzene, cobalt, and aspartame**


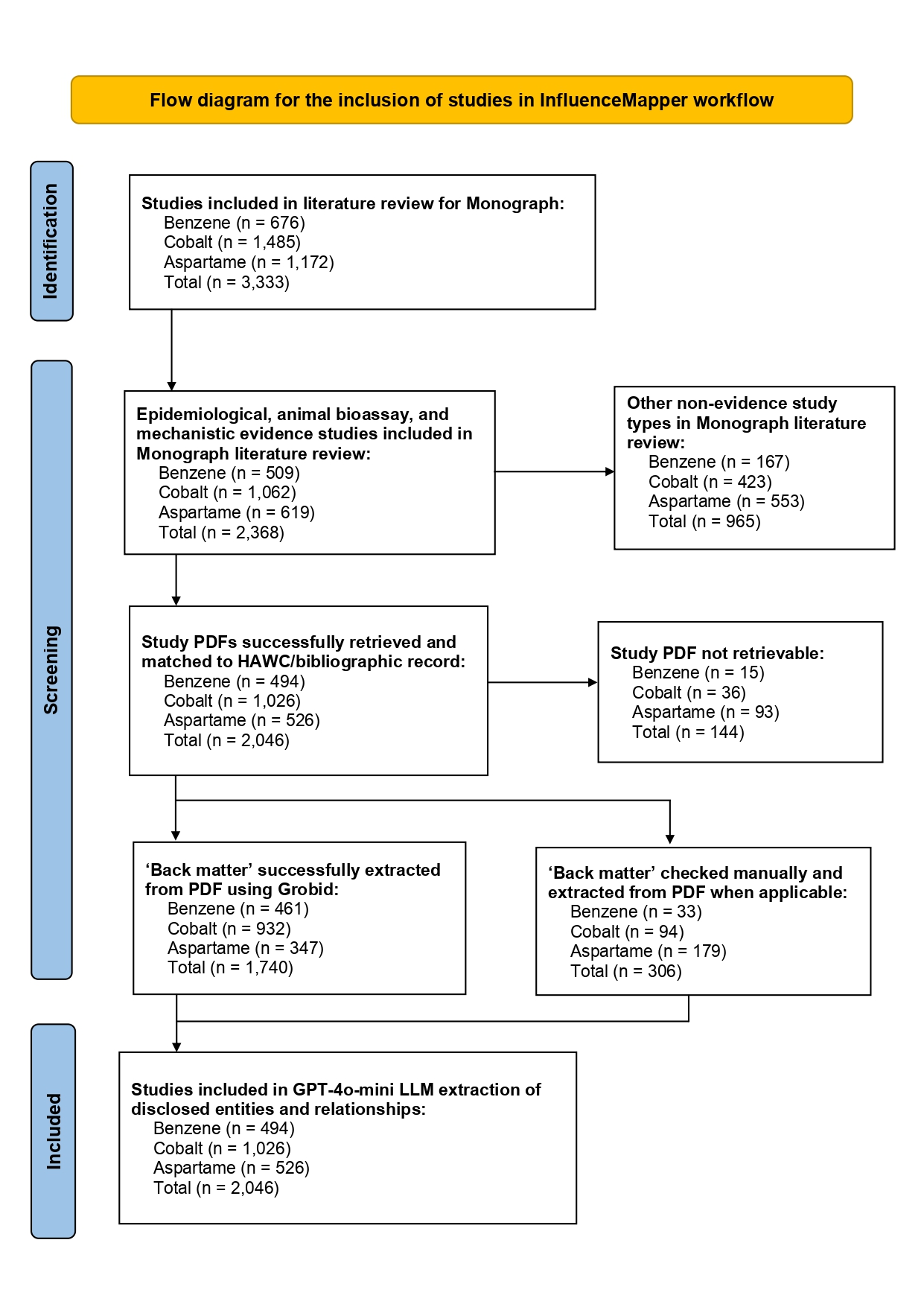


**Table S2. Keywords used to facilitate the identification of ‘industry or industry-funded entities’ from all disclosed entity names identified by InfluenceMapper in ‘back matter’ of published articles**

inc, llc, ltd, gmbh, company, companies, corporation, corp, co, producers, production, industry, manufacturers, plant, technologies, laboratories, consulting, consultants, refinery, refiners, factory, shipyard, insurance, biopharma, pharmaceuticals, therapeutics, pharma, benzene, american chemistry council, american chemical council, oil, petroleum, petro-, api, bp, shell, exxon, mobil, exxonmobil, esso, chevron, husky, neste, zeneca, coal, eni, total, totalenergies, statoil, centrica, sintef, engie, nofo, geocible, alion, nicolaides pathology, veritas, alion, pertra, norsk hydro, applied biosystems, pgs, norge, itri, inhalation toxicology research institute, morinaga, dow chemical, cobalt, antimony, tungsten, itia, nipera, clydach, ibm, eurometaux, inco limited, australian welding research association, johnson & johnson, toxys, biotronik, zimmer biomet, depuy, umicore, zelltek, roche, photocure, renascience, amersham, squibb, merck, boehringer, medtech, med-tech, edf, falconbridge, pfizer, eli lilly, amgen, genentech, sanofi, glaxo, glaxosmith, glaxosmithkline, janssen, novartis, abbott, nordisk, inquis, gilead, bayer, monsanto, takeda, searle, biomarin, bosch, novo nordic, novo nordisk, beverage, aba, sweeteners, sweetener, nutrasweet, isa, calorie control, ccc, sugar, pepsico, stevia, soy nutrition, carbohydrate, fruit juice, peanut, tree nut, canola, coca cola, coca-cola, welch, sinosweet, cargill, unilever, pepsi, nestle, danone, wawa dairies, northrop, monell, quest, tate ad lyle, medtronic, unico/primo, ab inbev, general mills, kellogg, quaker, foods, grain foods foundation, american society for nutrition, entrinsic, dried fruit council, ajinomoto, chemrisk, chem-risk, cardno, toxstrategies, exponent, stantec, biomatech, ipri, international prevention research institute, iom, institute of occupational medicine, westat, council for tobacco research, health effects institute, energy institute, international life sciences institute, international life science institute, ilsi, food information council, mccormick science institute, bain capital, rti international, research triangle institute, orthopedic research excellence fund, nakatomi foundation, lundbeck foundation, angpanneforeningens foundation, hamner institutes, scimetrika
